# Editorial: Advancing personalized diagnosis and treatment in Parkinson's disease: integrating biomarkers, neuroimaging, and artificial intelligence

**DOI:** 10.3389/fnins.2025.1734524

**Published:** 2025-11-27

**Authors:** Alice Maria Giani, Steven Shafeek, Elisa Tatti

**Affiliations:** 1 Nash Family Department of Neuroscience and Friedman Brain Institute, Icahn School of Medicine at Mount Sinai, New York, NY, United States; 2 Ronald M. Loeb Center for Alzheimer's Disease, Icahn School of Medicine at Mount Sinai, New York, NY, United States; 3 Department of Genetics and Genomic Sciences and Icahn Institute for Data Science and Genomic Technology, Icahn School of Medicine at Mount Sinai, New York, NY, United States; 4 Department of Molecular, Cellular and Biomedical Sciences, City University of New York, School of Medicine, New York, NY, United States

**Keywords:** Parkinson's disease, biomarkers, neuroimaging, artificial intelligence, personalized medicine, multimodal diagnostics, precision neurology, cognitive impairment

## Introduction

Once viewed primarily as a late-onset movement disorder, Parkinson's disease (PD) is increasingly recognized as a multisystem syndrome with motor and non-motor symptoms arising from diverse genetic, molecular, neurological, and environmental factors.

With the number of individuals affected more than doubled over the past three decades (Global Burden of Disease 2021) and the dramatic growing prevalence of early-onset forms (Li et al.), researchers and clinicians are exploring how to integrate epidemiological insights, lifestyle determinants, and emerging biomarkers to identify PD pathophysiology and build personalized approaches to early diagnosis and treatment.

This Research Topic, *Advancing personalized diagnosis and treatment in Parkinson's disease: integrating biomarkers, neuroimaging, and artificial intelligence*, brings together original studies and reviews that showcase innovative methodologies and clinical insights pointing the way toward integrated patient-specific care for PD (Table 1).

**Table 1:** Summary of the study design, hypothesis and results of the studies included in the Research Topic.

**Author**	**Study type**	**Population**	**Hypothesis**	**Main results**
**Epidemiological**				
Li et al.	Epidemiological study	Global EOPD cases aggregated from the GBD Study 2021	The global burden of EOPD has increased from 1990 to 2021.	Incidence, prevalence, and DALYs increased while mortality declined; men affected more; middle-SDI regions showed highest disability/mortality driven by population growth highlighting regions and populations for prioritized screening & resource allocation.
**Neuroimaging electrophysiological biomarkers**				
Song et al.	Neuroimaging study (fMRI)	*N* = 3453; 2,052 PD 1,401 HCs (72 datasets).	Heterogeneous ReHo findings on fMRI will reveal distributed PD-associated dysfunctional networks across sensory, motor, and attentional systems.	FCNM identified PD network overlap with visual (49.24%), somatomotor (32.35%), dorsal attention (44.49%), ventral attention (67.97%) networks. ReHo-derived network topography identifies targets for network-based biomarkers and therapies.
Xu T. et al.	Neuroimaging study (MRI)	*N* = 100, 51 LPD, 49 RPD	LPD and RPD show distinct cortical structural and network topology, as assessed using SBM and analyzed via SCN derived from MRI.	LPD had reduced cortical surface area in right supramarginal gyrus, right precuneus, left inferior parietal lobule, left lingual gyrus vs RPD. Precuneus cortical surface area correlated with the MMSE in LPD. Side-of-onset MRI features may inform lateralized prognosis and cognitive risk determination.
Wei et al.	Neuroimaging (fMRI)	*N* = 87; 58 PD (29 early PD, 29 middle-to-late PD) and 29 HCs	There are significant changes in brain functional network topology in PD at different disease stages.	PD-E & PD-M both reduced clustering and nodal centrality in temporal-occipital regions; increased centrality in default mode & frontoparietal control networks; left middle frontal gyrus & right temporal pole centrality correlated with motor severity/disease stage. fMRI network markers assist in disease staging and motor severity determination.
Zhou et al.	Neuroimaging (MRI)	*N* = 59; 19 DIP, 20 PD, and 20 HC.	Hippocampal subfield atrophy links with cognitive, depressive, and motor symptoms in DIP vs PD/HC.	DIP showed significant subfield atrophy vs HCs; UPDRS patterns correlated with non-motor symptoms and hippocampal volume. Hippocampal MRI subfields may help distinguish DIP neurobiology and inform prognosis.
Zhao Y. et al.	Neuroimaging (EEG)	*N* = 76; 44 PD, 32 HCs	Lower global PAF and regional alpha PSD distinguish PPD-COG from PD-NC.	Global PAF reduced in PD vs controls; PD-COG showed lower alpha PSD in parieto-occipital/posterior temporal regions correlating with MoCA; ROC identified P3/PZ/T6 alpha PSD as optimal discriminators. EEG PAF/alpha PSD may serve as diagnostic markers for PD-related cognitive decline.
**Genetic, molecular, and cellular biomarkers**				
Xu H.-L. et al.	Genetic biomarker study	*N* = 182 PD, 74 GG carriers, 108 GA/AA carriers	BST1 rs4698412 A-allele predicts faster motor progression in PD patients carrying the A-allele variant and GG homozygotes.	GA/AA carriers had a greater rate of UPDRS-III increase vs GG carriers, however, no MMSE difference in cognition. BST1 rs4698412 A-allele is a genetic prognostic marker for motor deterioration.
He et al.	Genetic biomarker study	*N* = 304 total; 197 PD and 107 age-matched HCs	Circulating Parkin and related biomarkers will distinguish PD from controls.	Parkin, Hcy, total protein, and urea discriminated against PD patients with PRKN mutations from healthy controls (AUC = 0.841); Parkin associated strongly with PD status (mediated by CEA & albumin). Blood Parkin and pathway signals offer diagnostic biomarkers insights.
Chen J. et al.	Neuroimaging (MRI)/neuro-physiology study	*N* = 136; 36 PD-EDS, 100 PD-non-EDS	Elevated plasma NfL mediates the link between cortical thinning and EDS severity in PD.	PD-EDS showed cortical thinning (left supramarginal gyrus and right postcentral region), weakened functional connectivity, and higher plasma NfL that mediated left Supramarginal Gyrus thickness. Plasma NfL is a monitoring/predictive biomarker linking structural MRI changes to EDS symptom severity.
Chen H. et al.	Neuro-physiological study	*N* = 61; 41 drug-naïve PD (19 PD-RBD, 22 PD-nRBD) and 20 HCs.	PD-RBD exhibits distinct serum metabolic signatures that can serve as diagnostic biomarkers.	PD-RBD showed CCM disruption in PPAR; distinct metabolite panels differentiated PD subgroups from HCs. Serum metabolite markers may be diagnostic and suggesting targets.
**Epidemiology, digital, and clinical tools**				
Zhao J. et al.	Cross-sectional study	*N* = 54,027; adults from NHANES from 2005 to 2020.	Higher SII associates with greater PD prevalence/risk.	SII correlated positively with PD prevalence; dose-response present; stronger association in women, < 60 y, non-smokers, drinkers, non-obese. SII may serve as a population-level risk/diagnostic indicator and target for immune-modulating prevention strategies.
Zhou and Cheng	Epidemiological study	*N* = 18,277; Adults ≥40 years and older from NHANES 2005–2018.	There is a relationship between cardiovascular health score measured byLE8 and PD.	PD prevalence 1.3% among study population. Moderate (50–79) & high (80–100) LE8 scores had lower odds of PD vs low (0–49); dose-response observed. Diet and glycemic health drove inverse association. Modifiable LE8 components may reduce PD risk and guide prevention.
Wang et al.	Cross-sectional study	*N* = 161 40 AD-MCI patients, 40 PD-MCI patients; 41 PD PD-NC patients; 40 NC	The dCDT could distinguish MCI profiles between AD-MCI and PD-MCI by quantifying visuospatial and executive function.	Significant difference in cognitive function between AD-MCI and PD-MCI populations observed using dCDT. Task performance score correlated with visuospatial/executive subtest score on the MoCA scale indicating the efficacy of the dCDT test to help differentiate AD-MCI and PD-MCI for targeted treatment planning.
Culicetto et al.	Systematic review	18 studies that described or investigated oculomotor function in PD patients.	Eye-tracking technology with ML and VR integration improves PD diagnostic and monitoring of cognitive and motor symptoms.	Eye-tracking metrics such as saccade velocity, fixation duration, and pupil size are correlated with disease severity. ML and VR-enhanced models improved diagnostic performance making eye-tracking a reliable monitoring tool with potential for clinical application.
Twala	Systematic review	127 studies on AI applications in PD diagnosis and treatment.	A multimodal AI framework will achieve high accuracy for early PD detection and treatment-response prediction.	AI-driven PD diagnosis has accuracy rates ranging from 78 to 96%. Experimental framework achieved 94.2% accuracy in early-stage PD detection with a strength in identifying subtle motor fluctuations, voice pattern recognition, and gait analysis. Multimodal AI can improve early diagnosis and personalized therapy.
**Interventions**				
Huang et al.	Neuro-physiological study	60 SPF NIH male mice (18–22g)	Galangin treatment will attenuate MPTP-induced neuroinflammation and motor deficits via PI3K/AKT autophagy.	Galangin improved motor coordination, reduced neuronal damage, enhanced antioxidant capacity, downregulated Beclin-1 autophagy markers via PI3K/AKT activation. Galangin is a preclinical candidate targeting autophagy/PI3K-AKT for neuroprotection in PD.
Deng et al.	Case report	*N* = 1 71-year-old male patient with advanced PD, right-sided tremor, left-sided rigidity and significant dyskinesia.	Combined DBS of the GPi and PSA is a viable treatment for patients with asymmetric and advanced PD.	UPDRS-III score decreased from 73 to 46 and H-Y stage improved from stage 4 to 2.5. Asymmetrically targeted dual-lead DBS PSA-GPi may be a viable strategy for patients with asymmetric PD symptoms.

PD, Parkinson's disease; DBS, Deep Brain Stimulation; GPi, Globus Pallidus internus; PSA, Posterior Subthalamic Area; UPDRS-III, Unified Parkinson's Disease Rating Scale, Part III (motor section); H–Y, Hoehn–Yahr staging scale; EOPD, Early-Onset Parkinson's Disease; GBD, Global Burden of Disease; DALYs, Disability-Adjusted Life Years; SDI, Socio-demographic Index; AD-MCI, Alzheimer's Disease with Mild Cognitive Impairment; PD-MCI, Parkinson's Disease with Mild Cognitive Impairment; PD-NC, Parkinson's Disease with Normal Cognition; NC, Normal Cognition; dCDT, digital Clock Drawing Test; MoCA, Montreal Cognitive Assessment; NHANES, National Health and Nutrition Examination Survey; LE8, Life's Essential 8 cardiovascular health score; ML, Machine Learning; VR, Virtual Reality; AI, Artificial Intelligence; MRI, Magnetic Resonance Imaging; DIP, Drug-Induced Parkinsonism; HC, Healthy Control; fMRI, functional Magnetic Resonance Imaging; ReHo, Regional Homogeneity; FCNM, Functional Connectivity Network Mapping; PAF, Peak Alpha Frequency; PSD, Power Spectral Density; PD-COG, Parkinson's Disease with Cognitive Impairment; ROC, Receiver Operating Characteristic curve; P3/PZ/T6, standard EEG electrode positions; LPD, Left-Onset Parkinson's Disease; RPD, Right-Onset Parkinson's Disease; SBM, Surface-Based Morphometry; SCN, Structural Covariance Network; MMSE, Mini-Mental State Examination; PD-E, Early Parkinson's Disease; PD-M, Middle-to-Late Parkinson's Disease; RBD, REM Sleep Behavior Disorder; PD-RBD, Parkinson's Disease with REM Sleep Behavior Disorder; PD-nRBD, Parkinson's Disease without REM Sleep Behavior Disorder; CCM, Central Carbon Metabolism; PPAR, Peroxisome Proliferator-Activated Receptor; BST1, Bone Marrow Stromal Cell Antigen 1; PRKN, Parkin gene; Hcy, Homocysteine; CEA, Carcinoembryonic Antigen; SPF, Specific Pathogen-Free (mice); NIH, National Institutes of Health; MPTP, 1-Methyl-4-phenyl-1, 2, 3, 6-tetrahydropyridine (a neurotoxin used in PD models); PI3K/AKT, Phosphatidylinositol-3-Kinase/Protein Kinase B signaling pathway; Beclin-1, autophagy-related protein; EDS, Excessive Daytime Sleepiness; NfL, Neurofilament Light chain; SII, Systemic Immune-Inflammation Index.

## Neuroimaging and electrophysiological insights into Parkinson's disease

One of the biggest challenges in moving toward personalized care in PD lies in its clinical heterogeneity. Patients differ not only in the motor symptoms they display but also in the occurrence of non-motor symptoms, such as cognitive and mood disturbances. Neuroimaging approaches have been providing a deeper understanding of the neural basis of PD and several contributions to this Research Topic used such techniques to explore PD heterogeneity.

Using MRI, Song et al. conducted the largest meta-analysis to date of regional homogeneity (ReHo) alterations, combining 72 datasets from over 2,000 patients with PD and 1,400 controls. This study revealed a distributed dysfunctional network, involving the visual, somatomotor, dorsal and ventral attention networks, confirming that PD pathology extends beyond dopaminergic circuits to large-scale networks.

Another study (Xu T. et al.) investigated hemispheric lateralization, a hallmark of asymmetric dopaminergic degeneration associated with distinct symptom profiles (Voruz et al., 2025). Compared to right-onset patients, left onset patients with PD (LPD) showed reduced cortical area in the right supramarginal gyrus, right precuneus, left inferior parietal lobule, and left lingual gyrus. In LPD, the right precuneus area positively correlated with MMSE cognitive scores, consistent with previous reports (Syrimi et al., 2017; Lee et al., 2015). At the network level, LPD patients exhibited altered topological organization, with increased path length and reduced small-world index, indicative of reduced efficiency.

Building on this network-level approach, another study examined different stages of PD using resting-state fMRI and graph theory analysis (Wei et al.). Both early- and middle-to-late-stage patients with PD showed reduced clustering coefficients, indicating decreased local networks' specialization. More advanced patients showed an overall decline in network efficiency, both locally and across the whole brain. Importantly, centrality within the left middle frontal gyrus and right middle temporal pole correlated with clinical measures of motor severity and disease stage.

Going beyond PD staging and subtyping, another critical challenge lies in distinguishing idiopathic PD from secondary conditions, such as drug-induced Parkinsonism (DIP). Zhou et al. found greater reduction in bilateral whole hippocampal volume and subfields atrophy in DIP patients compared to patients with PD and controls, which correlated with cognitive deficits, depressive and motor symptoms. However, DIP patients' lower MoCA cognitive scores may partially explain these volumetric differences, emphasizing the need to control for baseline cognitive functioning in future studies.

Non-invasive electrophysiological (EEG) studies added further insights. Zhao Y. et al. showed that cognitively impaired patients with PD displayed lower EEG alpha (8–13 Hz) power in parieto-occipital and posterior temporal regions compared to cognitively intact patients, which correlated positively with MoCA score and best differentiated PD patients with and without cognitive impairment.

These findings align with longitudinal studies showing that alpha slowing predicts progression to PD-dementia (Klassen et al., 2011; Olde Dubbelink et al., 2013), reinforcing its value as a biomarker. Importantly, advances in wearable EEG technology and artificial intelligence now make it feasible to translate these markers into home-based monitoring systems, enabling continuous and personalized assessment of the patients' brain activity and cognition outside the clinic (Sigcha et al., 2023).

Overall, these contributions highlight how neuroimaging techniques can capture several dimensions of PD heterogeneity, from volumetric changes to network dysfunction, lateralization, and EEG slowing. By linking structural and functional alterations to specific symptoms, these methods move beyond simple PD subtyping to identify patients at risk of rapid progression or cognitive decline, guide treatments, provide tools for monitoring disease trajectories, and enrich clinical trials with biologically defined subgroups, thereby accelerating the translation of precision medicine approaches into practice.

## Genetic, molecular and cellular biomarkers

Complementing neuroimaging and electrophysiological evidence, other contributions in this Research Topic highlight how genetic, fluid, and metabolomic biomarkers can refine PD diagnosis, staging, and phenotyping, and how they can be combined or mapped onto brain networks for improved personalized care.

A single-center longitudinal study (Xu H.-L. et al.) found that, compared to GG homozygotes, BST1 rs4698412 A-allele carriers had faster motor, but not cognitive, decline, mainly driven by rigidity and bradykinesia. This highlights a common PD-risk variant as a potential progression biomarker, useful for refining prognosis and optimizing trials for accelerated motor decline.

In the WPBLC cohort, He and colleagues found that elevated plasma Parkin in patients with PD had moderate diagnostic accuracy. Combining Parkin with homocysteine, total protein, and urea improved discrimination. Parkin correlated with blood α-synuclein oligomers and phosphorylated α-synuclein, but not with motor severity. Mediation analyses suggested partial effects via albumin and carcinoembryonic antigen, and transcriptomics pointed to PINK1-PRKN mitophagy and related metabolic pathways. Together, these data support a minimally invasive multi-analyte blood panel for PD (He et al.).

Another study integrated plasma neurofilament light (NfL) with cortical morphometry and connectivity and observed that patients with PD with excessive daytime sleepiness (EDS) show higher NfL, focal parietal thinning (left supramarginal gyrus; right postcentral gyrus), and reduced parietal-frontal coupling. NfL partially mediated the link between supramarginal thickness and EDS severity. These results support combined neuroimaging and plasma NfL biomarkers to clarify EDS mechanisms and track non-motor progression (Chen J. et al.).

Finally, blood metabolomics identified seven molecules distinguishing PD from controls and a distinct three-metabolite pattern specific to PD with REM sleep behavior disorder (PD-RBD). Pathway enrichment indicated disruption of central carbon metabolism in PD and inactivation of PPAR signaling in PD-RBD, supporting these metabolites as candidate (Chen H. et al.).

## Epidemiology, digital, and clinical tools

Beyond neuroimaging and molecular markers, population-based studies have also identified systemic biomarkers of PD. In a cross-sectional analysis of NHANES 2005–2020 data, Zhao Y. et al. found that higher systemic immune-inflammation index (SII) values, derived from routine blood counts, were linked to greater PD prevalence, particularly at higher SII levels. These findings highlight PD's multisystem nature and support immune-inflammatory markers as useful indicators.

Similarly, Zhou and Cheng reported that cardiovascular health, reflected by higher Life's Essential 8 (LE8), correlated with lower PD risk, suggesting that maintaining cardiometabolic health may help reducing disease risk.

Digital and AI-based tools are also advancing PD diagnostics. In a cross-sectional study, a digital clock drawing test (dCDT) was implemented to differentiate PD patients with mild cognitive impairment (MCI) from Alzheimer's disease (AD) (Wang et al.). Combined metrics separated AD-MCI from PD-MCI with high accuracy and the overall drawing score correlated with the MoCA visuospatial/executive subtest score, supporting dCDT's value in cognitive assessment.

Eye-tracking, increasingly integrated with machine learning (ML) and virtual reality (VR), is further refining PD phenotyping. In their systematic review, Culicetto et al. reported that oculomotor metrics, such as saccade velocity, fixation duration, and pupil size, correlated with disease severity, and that integrating these metrics with ML/VR pipelines improve diagnostic accuracy and scalability. Together, these advances position eye-tracking as a promising biomarker platform for motor and cognitive dysfunction in PD, though standardization across devices and protocols remains essential before clinical adoption.

Finally, Twala reviewed AI applications in PD diagnosis and treatment, reporting accuracies spanning 78–96% across modalities, with neuroimaging leading on mean accuracy and multimodal systems offering the best generalizability. Building on this, the study illustrates a novel multimodal AI framework that achieves 94.2% overall accuracy and strong early-stage PD detection and outperforms traditional clinical assessment methods (Twala). So far, validation of this model used a simulated PD cohort, hence real-world, multi-site studies are needed before clinical use.

## Interventions

Deeper insights into PD risk factors and progression paired with biomarker-guided patient stratification are paving the way for effective personalized therapies. This Research Topic illustrates also this bench-to-bedside trajectory through two complementary studies: a flavonoid-based neuroprotective approach in a mouse model (Huang et al.) and a dual-target deep brain stimulation in a patient with asymmetric motor features (Deng et al.).

Huang et al. demonstrated that galangin mitigated MPTP-induced dopaminergic neurodegeneration in mice, restoring striatal dopamine levels, reducing neuroinflammatory cytokines and α-synuclein accumulation, increasing antioxidant enzymes and improving motor performance. Mechanistically, galangin activated PI3K/AKT/CREB-BDNF signaling, inhibited Beclin-1-dependent autophagy, and preserved TH-positive neurons, indicative of its disease-modifying efficacy in preclinical models (Huang et al.).

Taking another innovative approach, Deng et al. reported the first documented case of combining posterior subthalamic area (PSA) and globus pallidus internus (GPi) deep brain stimulation (DBS) in a single PD patient with pronounced left-right asymmetry. The PSA is a tremor-responsive target, whereas the GPi is favored for treating rigidity and dyskinesia. A left PSA lead suppressed right-sided tremor, and a right GPi lead treated left-sided rigidity and dyskinesia. After six months of dual-target DBS, motor status improved and tremor frequency decreased, while cognition remained intact with no significant adverse effects. As a single case with short follow-up, these findings support feasibility of symptom specific dual-target neuromodulation for individualized therapy, although larger controlled studies are needed to establish effectiveness (Deng et al.).

## Conclusions

As PD emerges as a multifaceted disorder extending beyond dopaminergic degeneration, progress in care will depend on closer integration of technology and neurobiologically informed clinical practice. Collectively, the studies in this Research Topic show that combining biomarkers, advanced neuroimaging, and AI-driven analytics enables earlier diagnosis, personalized treatment, and improved monitoring of disease progression, indicating an ongoing shift toward precision care.
